# Self-perceived competence in early diagnosis of cervical cancer among recently graduated physicians from Lima, Peru

**DOI:** 10.1371/journal.pone.0203778

**Published:** 2018-09-12

**Authors:** Jessica Hanae Zafra-Tanaka, Marcia Esther Hurtado-Villanueva, María del Pilar Saenz-Naranjo, Alvaro Taype-Rondan

**Affiliations:** 1 CRONICAS Centro de Excelencia de Enfermedades Crónicas, Universidad Peruana Cayetano Heredia, Lima, Peru; 2 Gerencia Central de Promoción y Gestión de Contratos, Seguro Social de Salud – EsSalud, Lima, Peru; 3 Hospital Nacional Alberto Sabogal Sologuren, Seguro Social de Salud – EsSalud, Lima, Peru; 4 Unidad de Investigación para la Generación y Síntesis de Evidencias en Salud, Universidad San Ignacio de Loyola, Lima, Peru; National Cancer Institute, UNITED STATES

## Abstract

**Objective:**

To assess the prevalence of recently graduated physicians who perceived themselves as adequately competent to perform Papanicolaou (PAP), Visual Inspection with Acetic Acid (VIA), and Visual Inspection with Lugol´s Iodine (VILI); and study its associated factors, in Lima, Peru.

**Methods:**

This cross-sectional study evaluated recently graduated physicians from Lima, Peru. Physicians were considered to perceive themselves as adequately competent if they had answered, "agree" or "strongly agree" when asked if they were competent enough to perform these screening tests. To evaluate the associated factors, prevalence ratios (PR) were calculated using Poisson regressions with robust variance.

**Results:**

Only 367/432 (86.2%) physicians perceived themselves as adequately competent to perform PAP, 257 (60.5%) to perform VIA, and 247 (58.1%) to perform VILI. Physicians who performed their gynecology/obstetric clerkship at hospitals from the police or armed forces had a higher proportion of perceiving themselves as adequately competent to perform VIA and VILI.

**Conclusions:**

Nine out of ten physicians perceived themselves as adequately competent to perform PAP, while six out of ten to perform VIA or VILI. The health care system in which the physicians performed their clerkship was associated with the prevalence of adequate self-perceived competence for performing VIA and VILI.

## Introduction

Cervical cancer is the fourth most common cancer in women worldwide, and the second most common in developing countries [1]. Cervical cancer represents 7.5% of female mortality in the world, 85% of which is concentrated in developing countries [1]. In 2012, 43% of the women diagnosed with cervical cancer in the world died [2].

In 2013, the World Health Organization (WHO) and the Pan American Health Organization (PAHO) published the “Guidelines for screening and treatment of precancerous lesions for cervical cancer prevention” [3], which focuses on a test and treat strategy in order to achieve early diagnosis and timely treatment of these lesions. For low-income countries, these guidelines state that PAP is useful, but suggest that cervical cancer is routinely screened with Visual Inspection with Acetic Acid (VIA) instead of Papanicolaou test (PAP) and colposcopy, because VIA is less expensive, non-invasive, and requires little equipment and infrastructure [4]. In addition, another widely used technique that has the same benefits, and provides greater facility than PAP to distinguish cervical lesions is Visual Inspection with Lugol’s Iodine (VILI) [5].

Even though VIA and VILI are the screening tests with greater sensitivity and specificity usually used in the first level of care [6, 7], their precision is observer-dependent, so poorly trained observers often report a high number of false positive results [8]. For this reason, it is necessary to carry out continuous training and periodic evaluations of personnel competence in the realization of these tests.

However, few studies have evaluated the competency of health personnel to perform these tests: a study in Mexico [9] has evaluated the performance of the PAP using a checklist applied by experts who directly observed nurses and physicians while performing the procedure. Studies performed in physicians, nurses and midwives from Morocco [10]; and in nurses from Ghana, and Thailand [11] used similar methodologies to assess competencies in the performance of VIA. Another study carried out through a web portal that included a test with images of VIA and VILI, managed to include physicians, nurses, and midwives from more than 100 countries worldwide [12].

In Peru, cervical cancer was the leading cause of cancer in women accounting for 24.1% of the cases of cancer. In this context, recently graduated physicians who perform the social service are needed to perform diagnostic exams of cervical cancer (Mainly PAP, IVAA, and VILI) in the first level of care. However, we have not found studies that address the competence of these professionals to perform such screening tests.

Competencies may be assessed using several techniques [13] which evaluate different constructs [14]. Among these techniques, the study of self-perceived competencies -the idea that persons have about their own competencies to perform certain tasks- is of huge importance, given that it is closely related to their motivation and attitudes, which can directly influence on their decisions about performing or not certain task [14, 15]. In our context, Peruvian physicians performing social service who feel they lack the competencies to perform the diagnostic exams of cervical cancer can decide not to perform them, losing valuable opportunities for early diagnosis. This is why the evaluation of self-perceived competencies is of great importance in our context.

Thus, the aim of the present study is to assess the prevalence of recently graduated physicians who self-perceived as adequately competent to perform PAP, VIA, and VILI; and further, to study its associated factors, in Lima, Peru.

## Methods

### Study design and population

This cross-sectional study was held during April 2017, and evaluated recently graduated physicians who attended the “VI Convención Nacional SERUMS” organized by the Medical College of Peru. This event was held in Lima, the capital of Peru, which has the largest number of medical schools[16]. The objective of this event was to provide information about the Peruvian social service (SERUMS) to physicians who performed their undergraduate studies in Lima, and who were going to perform this social service in 2017.

The SERUMS is a social service that every health professional has to undergo in order to work at public institutions or to perform the medical residency programs in Peru [17]. This social service lasts one year and is performed at first level of care establishments located in rural and poor areas. During this service, physicians are in many cases in charge of performing medical routine procedures such as the diagnostic exams of cervical cancer (either PAP, IVAA, or VILI).

All the physicians who attended the “VI Convención Nacional SERUMS” were invited to participate in the study. Only those who gave their informed consent were included in the study. For the analysis, physicians who did not study at a medical school located in Lima, those who finished studying medicine before 2016, those who had previously studied midwifery, and those who had incomplete data for the variables of interest were excluded.

### Procedures

At the beginning of the event, the researchers briefly explained the study to the physicians and invited them to participate. Self-administered surveys were distributed to all physicians who agreed to participate and completed an informed consent form. Participants had time to respond the survey before the beginning of the event and during breaks. Researchers were present during the application of the surveys in order to answer any questions from participants.

The data were double entered by different researchers independently. A researcher who did not participate in this process crosschecked the double-entered data. When inconsistent data were found, the surveys were reviewed to correct the mistakes.

### Instrument

The researchers developed an *ad hoc* instrument that included sociodemographic characteristics and self-perceived competencies to perform diagnostic exams of cervical cancer (PAP, IVAA, and VILI). Two Peruvian gynecologists reviewed the surveys. Also, this instrument was piloted through the interview of recently graduated Peruvian physicians from outside of Lima, who did not participate in the study.

### Variables

The main variables were the self-perceived competencies to perform screening tests for cervical cancer: PAP, VIA, and VILI. These screening tests were chosen because they are tests with good sensitivity and specificity that are usually used in the first level of care [6, 7].

In order to evaluate self-perceived competence, physicians were asked to report how appropriate the following three statements were for them: "I am adequately competent to perform PAP during SERUMS,” “I am adequately competent to perform VIA during SERUMS,” and "I am adequately competent to perform VILI during SERUMS". For each of the statements, there were five possible answers were proposed: "Strongly disagree,” "Disagree,” "Neutral,” "Agree,” and "Strongly agree.” Physicians who responded "Agree" or "Strongly agree" were considered to perceive themselves as adequately competent.

Other variables evaluated were: sex, age in years (in tertiles), type of university in which the physicians went to medical school (public or private), and in which health care system the physicians performed the gynecology/obstetric clerkship (this is referred to the last year of undergraduate study, where an intensive gynecology/obstetric rotation is performed, usually for three months, by Peruvian medical students).

In Peru, health facilities belong to one of four health care systems: 1) Ministry of Health (MINSA), where people with few economic resources are usually cared for; 2) Social Security (EsSalud), where formal workers and their relatives are usually cared for; 3) Armed forces/police, where those who belong to the armed forces, police, or navy are cared for; and 4) private clinics, where those who decide to acquire a private insurance and can afford it are cared for. Each health care system could have distinct academic features for the undergraduate clerkship.

### Data analysis

Absolute and relative frequencies were used to describe categorical variables, while central tendency and dispersion measures were used for quantitative variables.

The possible associated factors to the self-perception of adequate competencies for the performance of each of the cervical cancer screening tests were evaluated through crude prevalence ratios (PR) and their 95% confidence intervals (95% CI), calculated using the Poisson regression with robust variance. The analyses were carried out using a level of significance of 5%, with the software Stata v14 (StataCorp LP, 2015. College Station, Texas, USA)

### Ethics

This study was approved by the IRB of the “Hospital Nacional Docente Madre-Niño–HONADOMANI” (RCEI– 40, Lima, Peru). The participation in this study was voluntary and anonymous. Every physician filled out an informed consent form before the administration of the survey.

## Results

A total of 520 physicians were surveyed, of whom 65 were excluded because they had attended a medical school outside of Lima, 17 because they had finished medical school before 2016, and 6 physicians because they had studied midwifery previously. Thus, 432 physicians were included in the analysis (83.1%). The population of recently graduated physicians included in the study represents 31.3% of all physicians graduated in 2016 at universities in Lima, Peru (Table 1).

**Table 1:** Recently graduated physicians included in the study versus all the physicians recently graduated from universities from Lima, Peru.

University	Physicians graduated during the year 2016*	Physicians recently graduated included in the study**
USMP	342	119 (34.8%)
UNMSM	147	68 (46.3%)
URP	177	63 (35.6%)
UCSur	150	56 (37.3%)
UPSJB	250	46 (18.4%)
UNFV	105	39 (37.1%)
UPCH	120	24 (20.0%)
UPC	77	13 (16.9%)
Total	1368	428 (31.3%)

Recently graduated: those who finished their studies in the year 2016.

USMP: Universidad San Martín de Porres, UNMSM: Universidad Nacional Mayor de San Marcos, URP: Universidad Ricardo Palma, UCSur: Universidad Científica del Sur, UPSJB: Universidad Privada San Juan Bautista, UNFV: Universidad Nacional Federico Villareal, UPCH: Universidad Peruana Cayetano Heredia, UPC: Universidad Peruana de Ciencias Aplicadas.

*Source: “Comité Médico Joven” of the Medical College of Peru

** four physicians did not mention their university

The median age of the physicians included in the analysis was 25 years (Interquartile range: 24 to 27), 243 (56.3%) were female, 321 (74.3%) have performed their undergraduate studies in a private university, and 288 (68.4%) had performed their gynecology/obstetric clerkship in MINSA, 61 (14.5%) in EsSalud, 61 (14.5%) in armed forces/police hospitals, and 11 (2.6%) in private clinics (Table 2).

**Table 2:** Characteristics from the recently graduated physicians who attended the “VI Convención Nacional SERUMS”, 2017 (n = 432).

Characteristics	N (%)
Age	
22 to 24 years	144 (34.3)
25 to 26 years	154 (36.7)
27 to 42 years	122 (29.1)
Gender	
Masculine	189 (43.8)
Femenine	243 (56.3)
University	
Public	111 (25.7)
Private	321 (74.3)
Health care system in which the physician performed the gynecology/obstetric clerkship	
MINSA	288 (68.4)
EsSalud	61 (14.5)
Armed forces/police	61 (14.5)
Private	11 (2.6)

MINSA: Ministry of Health from Perú

EsSalud: Social security

**Fig 1:**
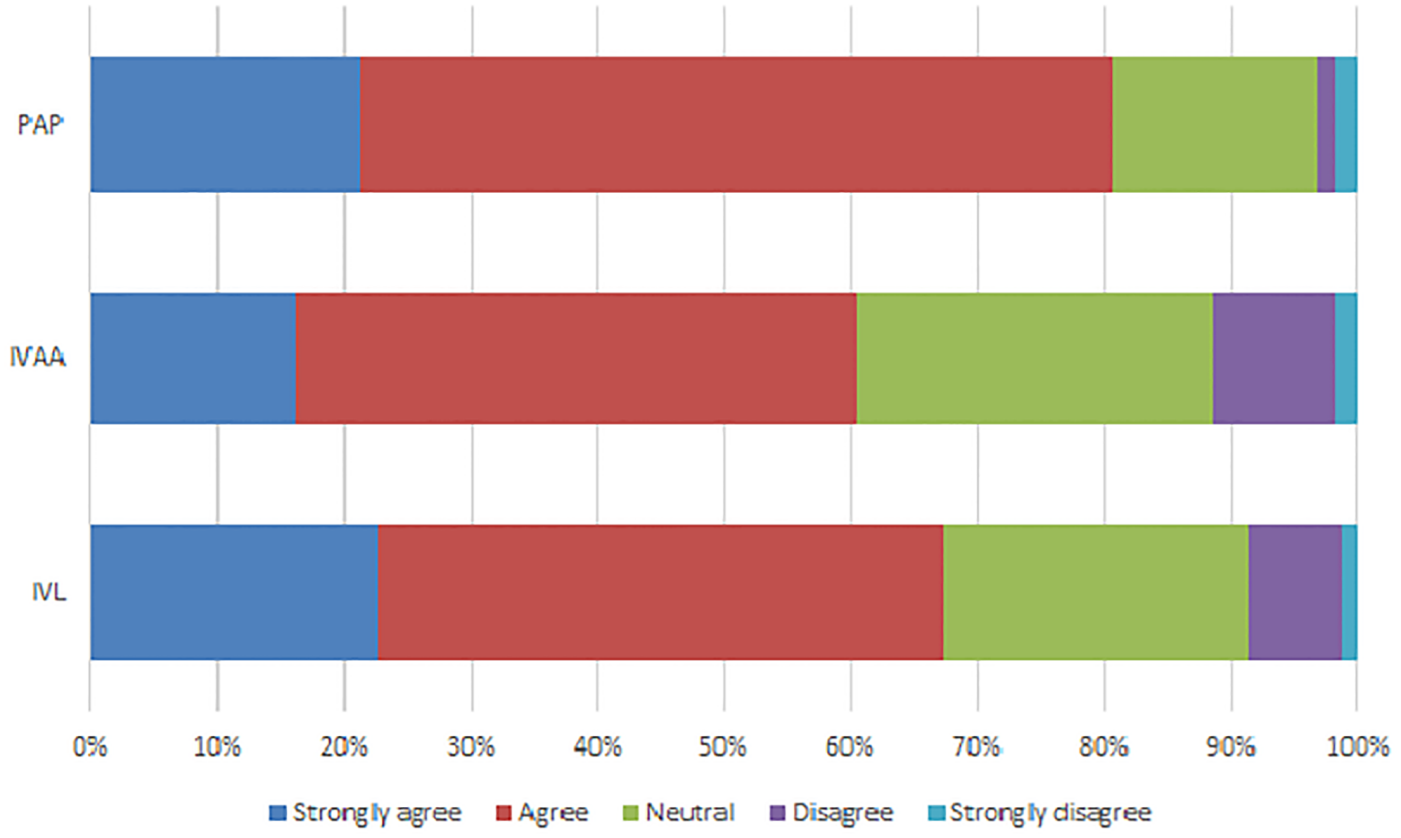
Self-perception of adequate competence to perform PAP, VIA and VILI. PAP: Papanicolaou, VIA: Visual Inspection with Acetic Acid, VILI: Visual Inspection with Lugol’s Iodine.

Regarding the proportion of physicians who perceived themselves as adequately competent to perform cervical cancer screening tests, 367 (86.2%) perceived themselves as adequately competent to perform PAP, 257 (60.5%) to perform VIA, and 247 (58.1%) to perform VILI (Fig 1).

Those who performed their gynecology/obstetric clerkship at the armed forces/police hospitals had a higher prevalence of self-perceived adequate competencies in VIA (PR: 1.32, 95% CI: 1.11–1.58) and VILI (PR: 1.31, 95% CI: 1.08–1.59) compared to those who performed their gynecology/obstetric clerkship in MINSA hospitals. (Table 3).

**Table 3:** Associated factors to self-perceived adequate competence to perform PAP, VIA and VILI.

Characteristics	Self-perceived adequate competence to perform…					
	PAP		VIA		VILI	
	n/N (%)	PR (IC95%)	n/N (%)	PR (IC95%)	n/N (%)	PR (IC95%)
Age						
22 to 24 years	127/143 (88.8)	ref	79/142 (55.6)	ref	86/143 (60.1)	ref
25 to 26 years	134/152 (88.2)	0.99 (0.91–1.08)	99/152 (65.1)	1.17 (0.97–1.41)	89/152 (58.6)	0.97 (0.81–1.18)
27 to 42 years	99/121 (81.8)	0.92 (0.83–1.02)	76/121 (62.8)	1.13 (0.92–1.38)	69/120 (57.5)	0.96 (0.78–1.17)
Gender						
Masculine	156/186 (83.9)	ref	117/186 (62.9)	ref	108/185 (58.4)	ref
Femenine	211/240 (87.9)	1.05 (0.97–1.13)	140/239 (58.6)	0.93 (0.80–1.09)	139/240 (57.9)	0.99 (0.84–1.17)
University						
Public	88/107 (82.2)	ref	59/107 (55.1)	ref	57/107 (53.3)	ref
Private	279/319 (87.5)	1.06 (0.96–1.17)	198/318 (62.3)	1.13 (0.93–1.37)	190/318 (59.7)	1.12 (0.92–1.37)
Health care system in which the physician performed the gynecology/obstetric clerkship						
MINSA	239/285 (83.9)	**ref**	161/284 (56.7)	**ref**	155/284 (54.6)	**ref**
EsSalud	54/59 (91.5)	1.09 (0.99–1.20)	37/59 (62.7)	1.11 (0.89–1.38)	35/59 (59.3)	1.09 (0.86–1.38)
Armed forces/pólice	55/60 (91.7)	1.09 (1.00–1.20)	45/60 (75.0)	**1.32 (1.11–1.58)**	43/60 (71.7)	**1.31 (1.08–1.59)**
Private	9/11 (81.8)	0.98 (0.73–1.30)	8/11 (72.7)	1.28 (0.88–1.87)	8/11 (72.7)	1.33 (0.91–1.94)

MINSA: Ministry of Health from Peru

EsSalud: Social security

PAP: Papanicolaou, VIA: Visual Inspection with Acetic Acid, VILI: Visual Inspection with Lugol’s Iodine

## Discussion

Nine out of ten recently graduated physicians perceived themselves as adequately competent to perform PAP, while six out of ten physicians felt adequately competent to perform VIA, and six out of ten felt adequately competent to perform VILI. It was found that the proportion of physicians who perceived themselves as adequately competent to perform VIA and VILI was greater for those who performed their gynecology/obstetric clerkship in the armed forces/police hospitals in comparison to those who performed their clerkship in MINSA hospitals.

Even though the competence of health professionals to perform screening tests for cervical cancer is important, we found only a few studies that have evaluated it. Most of them evaluated competence according to a checklist completed by specialists after the direct observation of the procedure [9–11], while one evaluated competence using an online test that consisted of clinical cases [12]. However, we did not find any study that has evaluated self-perceived competence.

Regarding PAP, we found that nine out of ten participants perceived that they were adequately competent to perform PAP. A study carried out in nurses, physicians, and interns at 21 first-level centers in Mexico during 2006 and 2007 evaluated personnel competence by direct observation. Experts were given a checklist, as the researchers were interested in determining the proportion of items out of this checklist that the health personnel performed correctly [9]. However, this study did not evaluate the proportion of physicians considered as competent to perform this procedure, so these results could not be compared with ours.

Regarding VIA, six out of ten physicians perceived themselves to be adequately competent to perform this test. A study carried out in Morocco on physicians, nurses, and midwives working in the first level of care evaluated competence to perform VIA by direct observation. Their objective was to assess the proportion of procedures in which the health personnel being evaluated performed all the recommended steps of a checklist [10]. However, this study does not present the proportion of physicians considered as competent, so its results could not be compared with ours.

Another study was conducted in Switzerland to assess the competency in differentiating between the normal cervix and cervical lesions with VIA and VILI. In order to achieve this, the researchers launched a web portal that included a free test consisting of 70 clinical cases with VIA and VILI images found that of 255 voluntary participants (including physicians, nurses, midwives, and medical students) from more than 100 countries, only two out of five participants passed the test, though this proportion was slightly higher among physicians [12]. This prevalence was lower than the prevalence of being self-perceived as competent for performing VIA/VILI in our study, although differences in the methodology prevents any comparison.

Although nine out of ten recently graduated physicians perceived themselves as adequately competent to perform PAP, only six out of ten perceived themselves as adequately competent to perform VIA or VILI. This could be because medical education in Peru is centered on the hospital environment, where PAP and colposcopy are the most common screening tests for cervical cancer, since hospitals have the professional and equipment necessary for the implementation of these tests. However, in the Peruvian first level of care, VIA and VILI would be more frequently used [3]. Thus, efforts should be made to teach Peruvian medical students competencies in not only hospital procedures, but also in those that they will perform as physicians at the first level of care, such as VIA and VILI.

On the other hand, health professionals working in first-level of care, especially in remote areas, should also be adequately trained in these procedures, for which virtual courses such as the one designed by the Geneva Foundation for Medical Education and Research [18], an open free virtual course, could be used. Additionally, it has been proposed that the use of Smartphones to take photos and consult in real time with a specialist in case of a doubt at the time of performing VIA or VILI could be useful in low-resource settings, although more research is needed to stablish a solid recommendation [19].

We found a higher prevalence of recently graduated physicians who perceived themselves as adequately competent in the realization of VIA and VILI among those who performed their gynecology/obstetric clerkship at the armed forces/police hospitals, compared to those who did at the MINSA hospitals. This may be due to the fact that a greater proportion of MINSA provided services are directed to the care of pregnant women and to the performance of obstetrics. Moreover, given the limited number of personnel, medical students could be programmed to rotate at the obstetric ward as the number of patients is higher than the one in the gynecology ward. However, the differences in training contents and in the supervision provided after training at different institutions might also influence in the self-perceived competencies of physicians. For this reason, the Superintendencia Nacional de Educación Superior Universitaria (SUNEDU), institution that supervises quality of education of Peruvian universities, should supervise each institution to secure and homogenize basic training in VIA/VILI performance.

One limitation of this study is that the level of competence was self-assessed, which is less reliable than direct observation of competence, performed by a group of experts because: 1) acquiescence bias might be present, meaning that the physicians included in the study tried to answer all the questions in a positive way, and 2) perceived competencies do not always match objectively measured competencies, as studies have found that physicians fail to rate themselves accurately [14, 20, 21]. The instrument used in this study directly asked the physician about his competence, while other studies have used objective assessments using check-lists. This check-lists can be developed according to manuals for performing VIA/VILI as the one created by the International Agency of Cancer [22]. However, self-perceived competencies are still important as self-perception of young physicians reflect subject’s motivation and can influence on their attitudes towards performing a test, and so influence on care decisions [14, 15].

Another limitation is the convenience sampling used, although we have no reasons to believe that there are great differences in the perception of competence between physicians who did and did not attend the event where the surveys were performed.

In spite of the limitations presented, this is one of the few studies that has assessed healthcare personnel competence in the realization of different cervical cancer screening tests, and provides results that are of interest for medical schools and for those in charge of the social service in Peru.

In conclusion, nine out of ten recently graduated physicians perceived themselves as adequately competent to perform PAP, while six out of ten perceived themselves as adequately competent to perform VIA or VILI. Having performed the gynecology/obstetric clerkship at an armed forces/police hospital was associated with a greater proportion of perception of adequate competence to perform VIA and VILI compared to those who performed their gynecology/obstetric clerkship at the MINSA. Future research should assess competencies of physicians using objective measures, such as simulations, clinical vignettes or tests containing photographs of cervical lesions so as to complement information.

## Supporting information

S1 File Minimal data set. (DTA)

S2 File Questionnaire used in the study. (DOCX)
