# Supplementary material for: Self-perceived competence in early diagnosis of cervical cancer among recently graduated physicians from Lima, Peru
Source: PLoS One. 2018 Sep 12;13(9):e0203778. doi: 10.1371/journal.pone.0203778 (PMC6135504; doi:10.1371/journal.pone.0203778)
Supplement: S2 File — (DOCX) [file pone.0203778.s002.docx]

**Versión en Español**

**Indicaciones:** Por favor marcar con una “x” [X] o llenar los espacios ___________ con letra clara, según sea el caso

**Variables sociodemográficas:**

1. Edad (en años): ________________

1. Sexo:

1 [ ] Masculino

2 [ ] Femenino

1. Estado civil actual:

1 [ ] Soltero

2 [ ] Casado

3 [ ] Conviviente

4 [ ] Otro _____________

1. ¿Usted terminó de estudiar otra carrera relacionada a salud previo al estudio de la carrera de medicina humana? Por favor indique cuál:

0 [ ] No he terminado otra carrera relacionada a salud

1 [ ] Sí, obstetricia

2 [ ] Sí, enfermería

3 [ ] Sí, psicología

4 [ ] Sí, otra (especifique cuál): ________________

1. Universidad donde realizó pregrado:

1 [ ] Universidad Nacional Mayor de San Marcos

2 [ ] Universidad Peruana Cayetano Heredia

3 [ ] Universidad Peruana de Ciencias Aplicadas

4 [ ] Universidad Nacional Federico Villareal

5 [ ] Universidad San Martín de Porres

6 [ ] Universidad Ricardo Palma

7 [ ] Universidad Privada San Juan Bautista

8 [ ] Universidad Científica del Sur

9 [ ] Univ Nacional José Faustino Sánchez Carrión (Huacho)

10 [ ] Otra. ¿Cuál?: _____________________________________________

1. ¿En qué ciudad realizó sus estudios de pregrado de Medicina Humana?

1 [ ] Lima

2 [ ] Otra: _______________________

1. ¿Usted ha realizado externado en áreas de medicina general o cirugía, en algún momento durante los últimos 3 años de la carrera? **(de manera EXTRACURRICULAR)**

1 [ ] No

2 [ ] Sí, pero menos de 2 meses en total (sumando todas las veces que realizó el externado)

3 [ ] Sí, de 2 meses o más

**Internado:**

1. ¿En qué año realizó el internado?

1 [ ] 2016

2 [ ] 2015

3 [ ] 2014

4 [ ] Antes del 2014

1. ¿En qué institución de salud realizó su internado? (puede marcar más de una alternativa, de ser el caso)

1 [ ] MINSA

2 [ ] EsSalud

3 [ ] Sanidades (policía, fuerzas armadas, naval)

4 [ ] Sector privado (clínicas)

1. ¿En qué sede hospitalaria ha realizado su internado? (Si en alguna rotación estuvo en más de una sede, colocar todas, y colocar primero aquella en el que haya pasado más tiempo. Si alguna rotación se realizó en una sede fuera de Lima, colocar la ciudad)
   1. Rotación de Medicina. Sede: ____________________________________________________________________________
   2. Rotación de Cirugía. Sede: ____________________________________________________________________________
   3. Rotación de Ginecología. Sede: ____________________________________________________________________________
   4. Rotación de Obstetricia. Sede: ____________________________________________________________________________
   5. Rotación de Pediatría. Sede: ____________________________________________________________________________
   6. Rotación de Neonatología. Sede: ____________________________________________________________________________
   7. Otra rotación: _____________________. Sede: ____________________________________________________________________________

**Competencias en ginecología:** Por favor, marque con una “X” según corresponda: **Poseo competencias idóneas para realizar estos procedimientos durante el SERUMS**

| ***Poseo competencias idóneas para realizar estos procedimientos durante el SERUMS*** | **1**  **Muy en desacuerdo** | **2**  **En desacuerdo** | **3**  **Neutro** | **4**  **De acuerdo** | **5**  **Muy de acuerdo** |
| --- | --- | --- | --- | --- | --- |
| 1. Toma de Papanicolaou (extracción de muestra cervical mediante un cepillo o paleta de ayre) |  |  |  |  |  |
| 1. Realización de Inspección visual con ácido acético (IVAA) |  |  |  |  |  |
| 1. Realización de inspección visual con lugol (IVL) |  |  |  |  |  |

Muchas gracias por su colaboración

**Version in English**

**Indications: Please mark with an "x" [X] or fill in the spaces ___________ with legible letters, depending on the case**

**Sociodemographic variables:**

1. Age (in years): ________________

1. Sex:

1 [ ] Male

2 [ ] Female

1. Current civil status:

1 [ ] Single

2 [ ] Married

3 [ ] Cohabiting

4 [ ] Other _____________

1. Did you finish studying another health career prior to studying human medicine? Please indicate which:

0 [ ] I have not finished another career related to health

1 [ ] Yes, midwifery

2 [ ] Yes, nursery

3 [ ] Yes, psychology

4 [ ] Yes, other (which?): ________________

1. University where you performed undergraduate:

1 [ ] Universidad Nacional Mayor de San Marcos

2 [ ] Universidad Peruana Cayetano Heredia

3 [ ] Universidad Peruana de Ciencias Aplicadas

4 [ ] Universidad Nacional Federico Villareal

5 [ ] Universidad San Martín de Porres

6 [ ] Universidad Ricardo Palma

7 [ ] Universidad Privada San Juan Bautista

8 [ ] Universidad Científica del Sur

9 [ ] Univ Nacional José Faustino Sánchez Carrión (Huacho)

10 [ ] Other. Which?: ___________________________________________

1. In what city did you do your undergraduate studies in Human Medicine?

1 [ ] Lima

2 [ ] Other: _______________________

1. Have you done externship in areas of general medicine or surgery, at any time during the last 3 years of the career? (in an EXTRACURRICULAR way)

1 [ ] No

2 [ ] Yes, but less tan 2 months in total (adding all the times you did the externship)

3 [ ] Yes, 2 or more months

**Internship:**

1. In what year have you performed internship?

1 [ ] 2016

2 [ ] 2015

3 [ ] 2014

4 [ ] Before 2014

1. In which health institution did your internship? (can mark more than one alternative, if applicable)

1 [ ] MINSA

2 [ ] EsSalud

3 [ ] Sanidades (policía, fuerzas armadas, naval)

4 [ ] Private institutions (“clínics”)

1. In what hospital has your internship been carried out? (If in any rotation it was in more than one location, place all of them, and clarify the one that has spent the most time in. If any rotation was made in a location outside of Lima, place the city)
2. Medicine rotation. Hospital: ____________________________________________________________________________
3. Surgery rotation. Hospital: ____________________________________________________________________________
4. Gynecology rotation. Hospital: ____________________________________________________________________________
5. Obstetrics rotation. Hospital: ____________________________________________________________________________
6. Pediatrics rotation. Hospital: ____________________________________________________________________________
7. Neonatology rotation. Hospital: ____________________________________________________________________________
8. Other rotation: ___________________. Hospital: ____________________________________________________________________________

**Competencies in gynecology:** Please mark with an "X" as appropriate: I have the skills to perform these procedures during the SERUMS

| I have the skills to perform these procedures during the SERUMS | **1**  **Strongly disagree** | **2**  **Disagree** | **3**  **Neutral** | **4**  **Agree** | **5**  **Strongly agree** |
| --- | --- | --- | --- | --- | --- |
| 1. Papanicolaou (cervical sample extraction by brush or air pallet) |  |  |  |  |  |
| 1. Performing visual inspection with acetic acid (VIA) |  |  |  |  |  |
| 1. Realization of visual inspection with lugol (IVL) |  |  |  |  |  |

Thank you very much for your collaboration
